# Correlation of serum vitamin D and IL-8 to stages of periodontitis: a case-control analysis

**DOI:** 10.1007/s00784-024-06025-2

**Published:** 2024-11-16

**Authors:** Wafaa Saleh, Fatma Ata, Nessma A. Nosser, Bassant Mowafey

**Affiliations:** 1 Oral Medicine, Periodontology, Diagnosis and Oral Radiology Department, Faculty of Dentistry, Mansoura University, Mansoura, 33516 Egypt; 2 Clinical Pathology Department, Faculty of Medicine, Mansoura University, Mansoura, 33516 Egypt

**Keywords:** Periodontitis, Vitamin D, Interleukin-8, Stages

## Abstract

**Objectives:**

The current literature lacks the correlation between serum levels of vitamin D and interleukin-8 (IL-8) to the stages of periodontitis. The present research objectives are to evaluate the serum levels of vitamin D and IL-8 in periodontitis participants and healthy controls and to measure their correlation with the stages of periodontitis.

**Methods:**

The current case-control study was conducted on patients with periodontitis and healthy controls. After obtaining a questionnaire from the participants, the following clinical parameters were measured; periodontal probing depth (PPD), clinical attachment loss (CAL), plaque index (PI), gingival index (GI), bleeding on probing (BOP), and tooth count. The serum levels of vitamin D and IL-8 were measured using ELISA kits. Then, we measured the correlation of the stages of periodontitis with the serum levels of vitamin D and IL-8.

**Results:**

Ninety-eight participants; 52 with periodontitis and 46 healthy controls were included. The patients with periodontitis showed a significantly lower level of vitamin D, higher PPD, CAL, BOP, and lower number of teeth than the controls. In addition, serum vitamin D significantly correlated with the stages of periodontitis. Serum IL-8 showed no significant difference between the study and control groups while it does not significantly correlate with the stages of periodontitis.

**Conclusion:**

The current study’s findings suggest a potential association between serum level of vitamin D with severity of periodontitis which necessitates screening vitamin D status in patients with periodontitis and investigating the possibility of vitamin D supplementation in decreasing the progression of periodontitis.

## Introduction

Periodontitis is a chronic inflammation that affects the tissues supporting the tooth. It represents a major global oral health challenge. Several microorganisms were reported to cause periodontal diseases resulting in an immune system that reacts to pathogens found in dental plaque, causing a series of inflammatory reactions [1, 2]. The main goal of the Periodontal therapy is to reduce the bacterial number and regulate the microbial stability towards a microbiota that fosters periodontal health. These alterations result in decreased gingival inflammation and a comparatively stable level of periodontal attachment [3, 4].

Multiple systemic diseases have been associated with periodontitis such as coronary heart disease and diabetes. However, other factors were associated with periodontitis, influencing the progression of periodontal diseases [5–7]. Vitamin D has an important function in regulating calcium and phosphorus metabolism and supporting the immune system. It is a fat-soluble vitamin available in certain foods and supplements and it can be produced in the skin after exposure to sunlight. Vitamin D has a protective effect through modulation of the inflammatory response in certain diseases including coronary heart diseases, diabetes mellitus, and cancer [8–10]. Decreased ingestion of vitamin D may result in diminished mineralization of bone, negative calcium imbalance, and weakening of the structure of bone. Children with vitamin D deficiency are diagnosed with rickets while adults deficient in vitamin D show signs of osteoporosis [11, 12].

Several research studies have suggested that vitamin D deficiency can be potentially linked to the progression of periodontal diseases [13–17]. The mechanism by which vitamin D can limit the progression of periodontitis can be explained through the antimicrobial and anti-inflammatory effects of vitamin D. In addition, it was reported that vitamin D receptors in the immune cells of the periodontal tissues can protect the endothelium of the tissues by decreasing the production of the inflammatory cells following the insult of periodontopathogenic bacteria [18, 19].

Periodontopathogenic bacteria in the gingival crevicular fluid stimulate the immune system and cause the production of proinflammatory cytokines. The level of the inflammatory cytokines can help as a diagnostic aid in evaluation of the severity of periodontal diseases [20–22]. Interleukin-8(IL-8) is a proinflammatory cytokine in periodontal diseases. Several cells produce IL-8 in response to microbial invasion in tissue such as macrophages, fibroblasts, and epithelial cells. It enhances the requirement of neutrophils from the highly vascular gingival tissue to the gingival crevicular fluid resulting in tissue inflammation and periodontal destruction [23, 24]. A large body of research has studied the relationship between IL-8 levels and periodontal diseases. While some studies reported higher levels of IL-8 in periodontitis patients [25–27], other studies detected opposite results [28–30].

A recent comprehensive meta-analysis established a considerable association between the frequency of periodontitis and the lower serum levels of vitamin D which suggest the periodontal outcomes can be improved by combination of vitamin D supplementation with scaling and root planning [31]. While these results show the value of vitamin D supplementation in controlling periodontal diseases, there remains a need to investigate the underlying mechanisms through which vitamin D deficiency affects periodontal disease progression.

Since the lower serum levels of vitamin D and raised levels of IL-8 have been linked to higher levels of inflammation, we aimed to address this gap by investigating the relationship between serum vitamin D and IL-8 across different stages of periodontitis, particularly using the recently developed classification of periodontitis which provide comprehensive categorization of periodontitis stages. We seek to provide targeted periodontal therapy based on the inflammatory profile of individuals.

## Methods

The current study was designed as a case-control study in which we matched the age and gender of the participants in the study and control groups. The matching process involved two steps; the first step was the selection of patients in the study group. The second step involved selection of participants of the control group to match the gender and closer ages to the participants with periodontitis.

The study was conducted on patients attending the Periodontology clinic at Mansoura University, Faculty of Dentistry where the local ethical committee approved the protocol of the study (IRB Approval Number: A24020822). All participants received a detailed explanation of the study protocol including the questionnaire, clinical evaluation, and laboratory investigation. Then, patients willing to continue the study provided informed consent for participation. The questionnaire was the first step in our study, and it included the following data: patient’s name, age, gender, educational level, and medical history.

### Inclusion and exclusion criteria

#### Inclusion criteria

##### Study group

The study group included patients diagnosed with periodontitis according to the recent classification of periodontal diseases [32]. The following specific inclusion criteria were applied as follows:

A. **Teeth count**: A minimum of twenty teeth were required for each patient to be included in the current study.
B. **Diagnosis of periodontitis**: Periodontitis was diagnosed according to the presence of interdental clinical attachment loss (CAL) at two or more non-adjacent teeth or buccal CAL at two or more teeth. Cases with CAL due to non-periodontal causes were excluded such as cervical caries, third molar extraction, an endodontic periapical lesion that drains through the periodontium in addition to CAL due to vertical root fracture.
C. **Staging of periodontitis in the study group**: Cases of the study group were categorized according to the stages of periodontitis [32]:

###### Stage I

This stage is known as a mild stage and it is characterized by the existence of probing depth of 4 mm or less, interdental CAL of 1–2 mm with no tooth loss due to periodontitis, with horizontal type bone loss. The quantity of radiographic bone loss is below 15% of root length (coronal third of the root).

###### Stage II

It is a moderate stage periodontitis which requires the presence of a probing depth of 5 mm or less, interdental CAL of 3–4 mm, radiographic bone loss between 15 and 33% of root length (coronal third) without missed teeth due to periodontal cause.

###### Stage III

This is a severe stage periodontitis in which bone loss extends to the middle or apical third of the root and the pattern of bone loss may be vertical. In addition, the probing depth is 6 mm or more and interdental CAL of 5 mm or more. The missing teeth due to periodontitis are four or fewer.

###### Stage IV

It is an advanced stage of periodontitis with widespread bone loss reaching the middle or apical third of the root and it may show furcation involvement. In addition, probing depth is 6 mm or more and interdental CAL of 5 mm or more. The missing teeth due to periodontitis are five up to 20 teeth.

##### Control group

The following criteria were applied to the participants of the control group.

- No systemic illness.
- PPD of 3 mm or less.
- No CAL.
- No signs of bone loss.
- Not currently on medications.
- BOP less than 10% of the mouth.

#### Exclusion criteria

The participants with the following criteria were excluded from our study; individuals receiving the following drugs: antibiotics, oral contraceptives, immunosuppressive drugs, or anti-inflammatory drugs within the three months before the study started. In addition, pregnant or lactating females, and diabetic patients were excluded. Patients with a history of drugs that may influence bone and mineral metabolism and/or periodontal health including bisphosphonates, corticosteroids, anticonvulsants, calcium channel blockers, phenytoin, or cyclosporine and hormone replacement therapy, and patients on multivitamins or food supplements containing vitamin D were excluded. Furthermore, individuals with malabsorption syndrome and chronic diarrhea were excluded.

##### Calculations of sample size

The sample size calculation of the current investigation was based on the study by Isola et al., 2020 [33]. The calculation was based on CAL which is one of the main outcome variables of the study. Several factors were considered during calculation including the 0.26 effect size for CAL, the 0.5 expected standard deviation, the 0.05 two-sided significance level, and the 80% intended power. In order to detect a significant effect, each group would demand about 44 individuals. Because of this, we divided our study into two groups (study and control groups). To ensure sufficient statistical power and account for possible patients’ dropouts, we increased the sample size to 45 patients per group.

### Selection of the participants

**Fig. 1:**
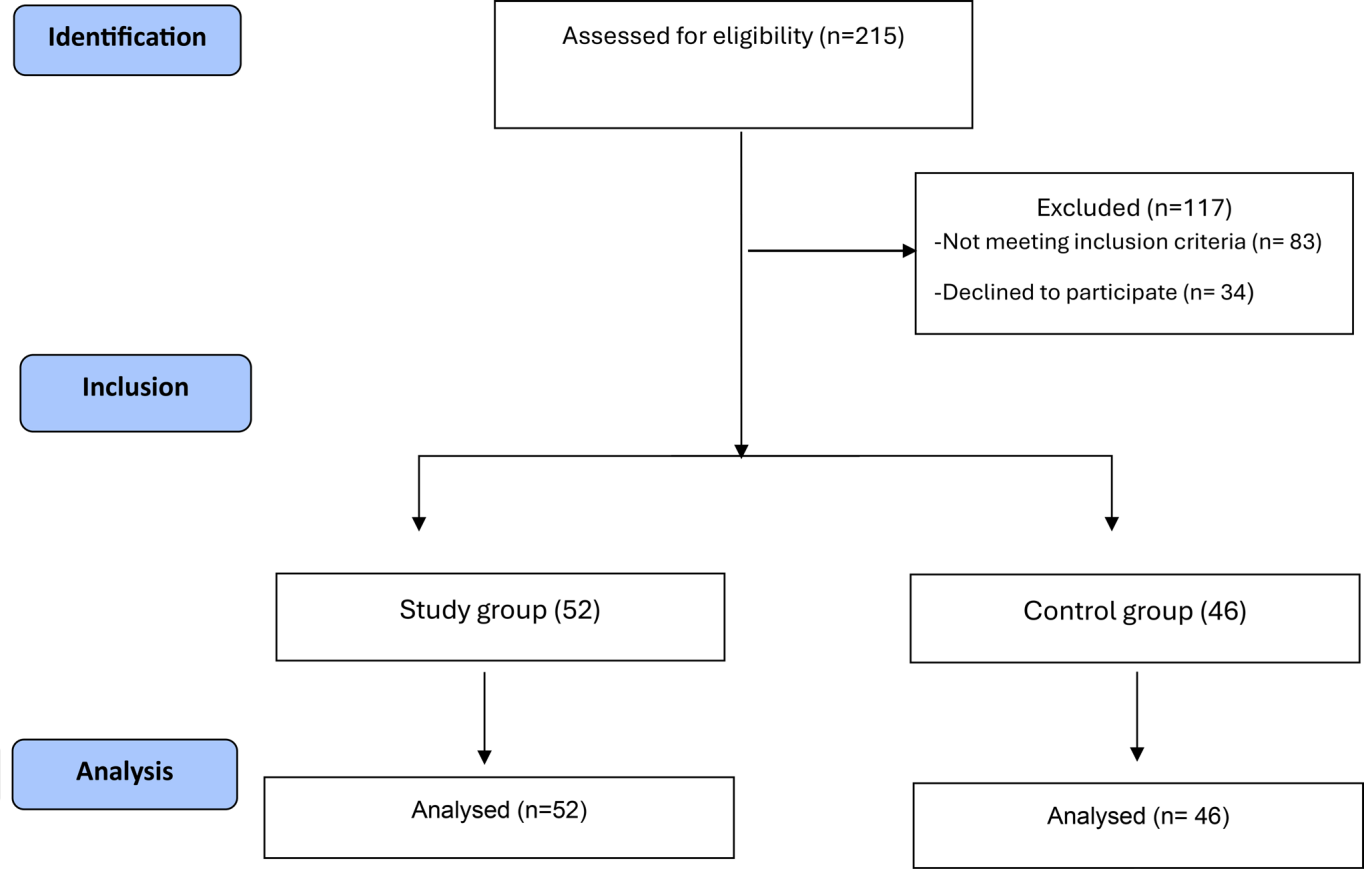
Flow diagram of the study

The participants of the current study were selected from the periodontology clinic in which 215 individuals were initially screened and evaluated according to the eligibility criteria. Eighty-three participants weren’t included due to the failure to meet the eligibility criteria. After discussing the study procedure with the participants, 34 individuals decided to withdraw from the study. Then 52 patients with periodontitis participated in the study group while the control group included 46 patients. Figure 1.

### Periodontal examination

Two qualified examiners (WS and FA) performed comprehensive full-mouth periodontal and radiographic examinations separately. The periodontal evaluation included measurements of PPD, CAL, plaque index (PI), gingival index (GI), and bleeding on probing (BOP).

### Measurement of the clinical parameters

#### A-Primary outcomes (PPD and CAL)

With the exception of the third molars, the primary outcomes were recorded from six locations per tooth: mesio-buccal, mid-buccal, disto-buccal, mesio-lingual, mid-lingual, and disto-lingual [34, 35]. At the six predetermined places, the PPD was measured in millimeters from the free gingival margin to the base of the gingival sulcus. We averaged the measurements obtained for the six areas surrounding each tooth to get the mean PPD for each tooth [35]. Then, at the six preset sites surrounding each tooth, CAL was measured from the cementoenamel junction to the base of the pocket. The CAL per tooth was calculated by averaging the six values [34]. We assessed PPD and CAL with the use of a graduated William’s periodontal probe.

#### B.Secondary outcomes

1. **Plaque index (PI)**: The PI was scored by visual inspection. The following scores were used to record the PI. Score(0) was given when there was no detected plaque; score(1) was given when there was a thin layer of plaque sticking to the free gingival margin and tooth surface; score(2) was recorded when there was a moderate buildup of plaque within the gingival sulcus and/or adjacent tooth surface, which can be perceived by the naked eye and score (3) was recorded if there is plenty of plaque within the gingival sulcus and/or on the gingival margin and adjacent tooth surface. For each tooth, four surfaces were scored according to the previous scoring system (Buccal, lingual, mesial, distal).T0 calculate the total PI for each participant, PI scores of all examined teeth were totaled and divided by the number of examined teeth [36].
2. **Gingival index (GI)**: It was used to measure the extension of gingivitis. Scoring of GI was based on several factors including the bleeding with probing, gingival texture, and contour of the gingiva. PI includes 4 different scores as follows; Score (0) was given when the examination showed the normal features of healthy gingiva; score (1) was recorded when the gingiva showed mild features of inflammation with slight edema and change of color without bleeding on probing; Score (2): indicates the signs of moderate gingival inflammation as redness, edema, with bleeding on probing. Score (3) was recorded when the gingiva showed the features of severe inflammation with ulceration, marked edema, and dark redness. In addition, this score is characterized by continuous gingival bleeding [36, 37].
3. **Bleeding on probing (BOP)**: The gingival sulcus was gently probed around each tooth to detect the presence of BOP using a Williams periodontal probe. The tooth was scored(0) or(1) according to the absence or presence of BOP respectively [38].
4. **Tooth loss due to periodontitis**: The data about missing teeth due to periodontal causes were obtained during interviewing the patients while the observed mobility of the teeth with difficulty in chewing were recorded.

All the primary and secondary outcomes were evaluated independently by two investigators (WS&FA).

### Inter-examiner and intra-examiner reproducibility of the clinical measurements

After the two trained examiners (WS&FA) measured the primary and secondary outcomes for the same patients independently, the inter-examiner reproducibility was measured. A moderate to high agreement was detected between both examiners (86.1%) with Cohen’s kappa coefficient (k) calculated at 0.64. The third examiner (BM) was consulted to solve the disagreement between WS&FA. In addition, the study outcomes were measured twice by the same examiner (WS) at two separate points in time. Then, we measured the intra-examiner agreement for CAL and PPD scores which showed agreement of CAL and PPD, 0.89 and 0.87 respectively.

### Laboratory assessment of serum levels of vitamin D and IL-8

Blood samples were withdrawn from the antecubital vein in the morning using 4 ml vacutainer glass tubes (BD Vacutainer, Becton, Dickinson and Company, USA). These samples were promptly transported to the laboratory, where they underwent centrifugation. The serum was preserved at − 20 °c till the time of analysis. Assaying vitamin D total and IL-8 was done in the Clinical Pathology Department, Faculty of Medicine, Mansura University. The total Vitamin D level was measured using the DRG 25-OH Vitamin D Total ELISA Kit based on competitive binding (DRG 25-OH Vitamin D Total ELISA Kit (DRG International Inc., USA; Order No. EIA-5396)). For the quantitative determination of Human IL-8 concentrations, a Sandwich ELISA method was employed, sourced from a kit from SinoGeneclon Co., Ltd. (China; Catalog number: SG-10269).

#### Statistical analysis of the data

Data were analyzed using IBM SPSS software version 20.0. The Chi-square test was used to examine correlations between categorical variables. When over 20% of cells had an expected count of less than 5, the Monte Carlo adjustment test was used. The normality of continuous data was measured using the Shapiro-Wilk and Kolmogorov-Smirnov tests. The student’s t-test compared normally distributed quantitative data between two groups, while the Mann-Whitney test compared non-normally distributed quantitative data between two groups. Results were considered significant at the 5% level.

## Results

In our study, we evaluated the sociodemographic variables of the participants. Fifty-two participants from the study group and 46 participants from the control group were analyzed. Both groups were matched in terms of age (*p* = 0.756). The data show similar gender distribution across the groups with no substantial difference (*p* = 0.37).

The comparison of the education level among the two studied groups showed a significant difference in education level (χ² = 8.402, *p* = 0.015), with a remarkably higher percentage of participants having a primary school education in the study group compared to the control group (44.2% vs. 21.7%). On the other hand, a higher proportion of participants with a university/academic education were found in the control group compared to the study group (34.8% vs. 13.5%). Table 1.

### Evaluation of the periodontal parameters

Measuring the BOP among the participants in the study and control groups showed that all patients in the study group had BOP while 45.7% of the control group had gingival BOP. In addition, we detected higher scores of PI and GI among the study group participants than the control group (*p* < 0.001). Furthermore, the study group showed higher PPD (4.85 ± 1.16 mm) compared to the control group (1.85 ± 0.76 mm) (*p* < 0.001). Evaluation of the number of teeth in both groups showed that the control group had a significantly higher number of teeth than the study group (*p* < 0.001). Table 1.

**Table 1:** Comparison of the clinical, demographic and laboratory parameters between the study and control groups

	Study group (*n* = 52)	Control group (*n* = 46)	Test of Significance	*p*
Gender				
Male	30(42.3%)	30 (65.2%)	χ^2^= 0.308	0.37
Female	22(57.7%)	16 (34.8%)		
Age (years)				
Mean ± SD.	40.30 ± 7.21	35.92 ± 7.08	t =-0.31	0.756
Education level				
Primary school	23 (44.2%)	10 (21.7%)	χ^2^= 8.402^*^	0.015^*^
High school	22 (42.3%)	20 (43.5%)		
University/academic	7 (13.5%)	16 (34.8%)		
Number of teeth				
Mean ± SD.	25.73 ± 2.98	30.50 ± 1.38	t = 10.344^*^	< 0.001^*^
Median (Min. – Max.)	26.0 (21–32)	31.0 (28–32)		
Bleeding on probing				
No	0 (0%)	25 (54.3%)	37.939^*^	< 0.001^*^
Yes	52 (100%)	16 (34.7%)		
Plaque index				
Score 0	0 (0%)	13 (28.3%)	χ^2^= 81.785^*^	< 0.001^*^
Score 1	1 (1.9%)	25 (54.3%)		
Score 2	5 (9.6%)	8 (17.4%)		
Score 3	46 (88.5%)	0 (0%)		
Mean ± SD.	2.87 ± 0.40	0.89 ± 0.67	t = 17.367^*^	< 0.001^*^
Median (Min. – Max.)	3 (1–3)	1 (0–2)		
Gingival index				
Score 0	0 (0%)	11 (23.9%)	χ^2^= 78.728^*^	< 0.001^*^
Score 1	0 (0%)	23 (50%)		
Score 2	8 (15.4%)	12 (26.1%)		
Score 3	44 (84.6%)	0 (0%)		
Mean ± SD.	2.85 ± 0.36	1.02 ± 0.71	t = 15.614^*^	< 0.001^*^
Median (Min. – Max.)	3 (2–3)	1 (0–2)		
Probing depth				
Mean ± SD.	4.85 ± 1.16	1.85 ± 0.76	t = 14.914^*^	< 0.001^*^
Median (Min. – Max.)	5.0 (3–8)	2.0 (1–3)		
Serum levels of Vitamin D level				
Severe deficiency (< 10.0 ng/mL)	23 (44.2%)	13 (28.3%)	χ^2^= 10.583^*^	^MC^p= 0.010^*^
Deficiency (10.0–19.9 ng/mL)	13 (25.0%)	21 (45.7%)		
Insufficiency (20.0–29.9 ng/mL)	16 (30.8%)	8 (17.4%)		
Sufficiency (≥ 30.0 ng/mL)	0 (0%)	4 (8.7%)		
Mean ± SD.	12.24 ± 8.43	15.68 ± 7.84	U = 1026.0	0.015
Serum level of IL-8				
Mean ± SD.	361.6 ± 359.7	356.1 ± 238.5	U = 892.0	0.46

U: Mann Whitney test

MC: Monte Carlo

### Analysis of serum levels of vitamin D and IL-8

The serum levels of vitamin D showed a significant difference between the two groups. The mean vitamin D level in the study group was less in comparison to the control group. Furthermore, higher percentage of patients in the study group had severe vitamin D deficiency (< 10.0 ng/mL) than the participants in the control group (44.2% vs. 28.3%), whereas a higher percentage of participants in the control group had sufficient vitamin D levels (≥ 30.0 ng/mL) (8.7%). No participant in the study group had sufficient level of vitamin D. We found no difference in the level of IL-8 between the study and control groups. Table 1.

### Evaluation of stages of periodontitis among the study group

The majority of cases in the study group showed advanced stages of periodontitis with 71% of the participants diagnosed with stage III or stage IV periodontitis while the mean CAL among the study group was 5.52 ± 2.05 mm. Table 2.

**Table 2:** Distribution of the participants in the study group according to stage of periodontitis and clinical attachment loss in study group (*n* = 52)

	No. (%)
Stages of periodontitis	
Stage I	2 (3.8%)
Stage II	12 (23.1%)
Stage III	18 (34.6%)
Stage IV	20 (38.5%)
Clinical attachment loss (mm)	
Mean ± SD.	5.52 ± 2.05
Median (Min. – Max.)	5 (2–11)

### Clinical and demographic characteristics of the study group in relation to stages of periodontitis

In the study group, sociodemographic and biomedical parameters were analyzed based on periodontitis stages. Gender distribution was similar across stages, with no significant difference. Mean age increased slightly with periodontitis severity but was not statistically significant. Education level didn’t correlate significantly with periodontitis stages; primary school education proportion remained consistent, as did high school or university/academic education proportions across stages. Table 3.

### Correlation between stages of periodontitis and serum levels of vitamin D and IL-8 in the study group

We detected a significant correlation between serum vitamin D levels and periodontitis stages (MCp = 0.018). Participants with Stage IV periodontitis (60%) were more likely than those in other stages to have a severe vitamin D deficiency (< 10.0 ng/mL). On the other hand, participants in Stage III had the greatest frequency of vitamin D deficiency (20.0–29.9 ng/mL) (50%). In all stages, no participant had sufficient levels of vitamin D (≥ 30.0 ng/mL). On the contrary, we found no significant association between the severity of periodontitis and IL-8 levels. Table 3.

**Table 3:** Demographic, clinical and laboratory characteristics of the study group in relation to the stages of periodontitis

Study group (*n* = 52)	Stages of periodontitis				Test of significance.	*P* value
	Stage I (*n* = 2)	Stage II (*n* = 12)	Stage III (*n* = 18)	Stage IV (*n* = 20)		
Gender						
Male	1 (50%)	5 (41.7%)	8 (44.4%)	8 (40%)	χ^2^ = 0.471	^MC^*p*=1.000
Female	1 (50%)	7 (58.3%)	10 (55.6%)	12 (60%)		
Age (years)						
Mean ± SD.	46.0 ± 5.66	45.67 ± 15.16	42.11 ± 7.24	48.90 ± 11.08	F = 1.208	0.317
Males	50	49.23 ± 16.9	44.05 ± 3.21	50 ± 5.03		
Females	42	42.44 ± 15.7	40.06 ± 4.03	46 ± 6.05		
Education level						
Primary school	1 (50%)	6 (50%)	8 (44.4%)	8 (40%)	χ^2^ = 5.441	^MC^*p*=0.484
High school	0 (0%)	4 (33.3%)	7 (38.9%)	11 (55%)		
University/academic	1 (50%)	2 (16.7%)	3 (16.7%)	1 (5%)		
Serum level of Vitamin D						
Severe deficiency (< 10.0 ng/mL)	1 (50%)	7 (58.3%)	3 (16.7%)	12 (60%)	χ^2^= 13.398^*^	^MC^p= 0.018^*^
Deficiency (10.0–19.9 ng/mL)	1 (50%)	4 (33.3%)	6 (33.3%)	2 (10%)		
Insufficiency (20.0–29.9 ng/mL)	0 (0%)	1 (8.3%)	9 (50%)	6 (0%)		
Sufficiency (≥ 30.0 ng/mL)	0 (0%)	0 (0%)	0 (0%)	0 (0%)		
Mean ± SD.	12.15 ± 6.86	11.32 ± 6.94	17.31 ± 7.76	12.76 ± 9.46	H = 5.888	0.117
Median (Min. – Max.)	12.2(7.3–17)	8.65 (1–22)	16.50 (6–29)	8.1 (4.5–28)		
Serum level of IL-8						
Mean ± SD.	280.5 ± 27.58	294.3 ± 41.51	489.9 ± 598.8	294.6 ± 40.31	H = 0.414	0.937
Median (Min. – Max.)	281 (261–300)	301(210–352)	282 (236–2130)	288 (247–380)		

MC Monte Carlo, H: H for Kruskal Wallis test

F F for One way ANOVA test

The serum Vitamin D levels demonstrated a significant positive correlation with both PPD and CAL (*P* ≤ 0.05). However, serum vitamin D levels did not significantly correlate with PI, GI, teeth count, or serum IL-8 level (*P* > 0.05). Furthermore, there was no marked correlation between the serum level of IL-8 and PI, GI, PPD, CAL, or teeth count. (*P* > 0.05). Table 4.

**Table 4:** Correlation of the clinical and biomedical parameters in the study group

Study group (*n* = 52)	Serum level of Vitamin D		Serum level of IL-8	
	*r*_s_	*p*	*r*_s_	*p*
Plaque index (PI)	-0.121	0.391	0.185	0.189
Gingival index (GI)	-0.252	0.071	0.116	0.415
Periodontal probing depth (PPD)	0.29	0.03	0.116	0.413
Clinical attachment loss (CAL)	0.36	0.01	-0.039	0.782
Number of teeth	0.234	0.094	-0.040	0.780
Serum level of vitamin D			-0.084	0.555

r_S_: Spearman coefficient

## Discussion

Understanding the correlation between serum levels of vitamin D and IL-8 to the severity of periodontitis can play an outstanding role in prevention and treatment of periodontitis [16, 18, 39, 40]. We aimed to shed the light on the possible inflammatory correlation between serum levels of vitamin D and IL-8 to the progression and severity of periodontitis. To the best of our knowledge, this is the first study to offer a comprehensive combined analysis of serum vitamin D and IL-8 levels in relation to the stages of periodontitis classified according to the new classification of periodontal diseases [32]. For the aim of validity, this study excluded individuals with diabetes mellitus (DM), and smokers. DM patients were not included due to the intricate relationship between diabetes and periodontitis [41, 42]., while smokers were excluded to prevent residual confounding factors associated with smoking [43].

In the current study, we matched the age and gender of the participants in both groups to make sure that both the age and gender do not act as confounding factors that might affect the results of both groups, enabling a more precise evaluation of the correlation between periodontitis and blood biomarkers.

The socioeconomic status of the individuals may affect their periodontal health. We noticed that the highest percentage of participants in the control group showed a higher level of education. On the other hand, in the study group, we found a higher percentage of patients with lower levels of education. Several factors may emphasize these outcomes in both groups including the lower economic levels, decreased accessibility to oral health care, improper diets, decreased oral hygiene measures, and smoking [44–46] Similarly, in a study conducted by Celeste et al.2019, they measured the correlation between the severity of periodontitis with the education levels among 10,000 participants. They found a negative linear relationship between both conditions in which higher education levels were associated with the lower severity of periodontitis [46].

Tooth loss acts as a severe outcome of periodontitis while it indicates the rate of progression of the disease and the effectiveness of earlier periodontal therapies [47, 48]. When compared to the control group, the study group’s tooth count was noticeably lower. Helal et al.2019 systematically analyzed the predictors of teeth loss in patients with periodontitis. They analyzed the data for patients who received active periodontal treatment and supportive periodontal therapy for three years. They demonstrated that higher PPD, mobility, furcation involvement in molars, the presence of IL-1 polymorphism, and higher bone loss were all associated with the raised risk of tooth loss [49].

Evaluation of serum vitamin D levels in the current study showed that patients with periodontitis had lower levels of vitamin D than the controls. In agreement with our findings, other studies reported that patients with periodontitis had significantly lower levels of vitamin D than the healthy controls [50, 51]. In addition, a recent cross sectional study included 2,928 participants reported a negative association between the severity of periodontitis and the serum levels of vitamin D [52]. However, other studies did not find any association between vitamin D levels and periodontal diseases [53–55]. Several factors can induce vitamin D deficiency including systemic health problems affecting the metabolism of vitamin D, limited sun exposure and dietary deficiency [13, 14, 16, 17].

Evaluation of the primary outcomes (PPD&CAL) showed significant correlation with the lower levels of vitamin D among the study group participants. Similarly, the severe CAL was related to the low serum levels of vitamins [18, 33, 56, 57]. Moreover, Isola et al.2020 reported that individuals with chronic periodontitis showed remarkably fewer number of teeth and higher PPD, CAL, and BOP compared to those in the healthy control group (*P* < 0.001) [33]. However, another study reported no correlation between serum 25(OH)D levels and CAL, PPD, and BOP in patients with periodontitis [58].

The current study analyzed the clinical and demographic characteristics in relation to periodontitis stages. We noticed a non-significant increase in mean age with more severe periodontitis. This suggests periodontitis may worsen with age due to cumulative effects, but age alone is not strongly correlated with severity. Other factors like oral hygiene, systemic health, and lifestyle choices also play crucial roles in periodontitis severity [59–62]. In addition, we found that the distribution of education levels did not show a significant correlation with the stages of periodontitis (MCp = 0.484). This suggests that educational attainment, in this study group, does not significantly influence the severity of periodontitis.

Menopause is believed to be a risk factor in many chronic diseases, especially periodontitis, which is further complicated by vitamin D deficiency. Menopausal women suffer from vitamin D deficiency due to several factors including lifestyle, limited sun exposure, and improper diet [63–65].

The association between vitamin D deficiency and periodontitis is not confined to menopausal or older populations. While the mean age of females in the study group was approaching the perimenopausal age which is younger than the menopause stage, we found that vitamin D deficiency remains a substantial factor influencing periodontal health across different age groups not just the menopausal stage which necessitates the assessment of vitamin D in both younger and older individuals. In addition, we found mixed results in studies conducted on postmenopausal women. In their study, Millen et al. examined postmenopausal women with an average age of roughly 66.6 years. They discovered no correlation between vitamin D levels and the prevalence of periodontal diseases, which was measured by tooth loss and alveolar crestal height [56]. On the other hand, women with periodontal diseases had decreased serum vitamin D in two prior case-control investigations, one on postmenopausal women with osteoporosis [66]and the other on pregnant women [67].

Analysis of the correlation between the stages of periodontitis to the serum levels of vitamin D is one of the main objectives of the current study. We noticed a significant association of the lower levels of vitamin D with the more severe stages of periodontitis. We found that 60% of patients with stage IV periodontitis had a severe vitamin D deficiency. A previous study investigated the correlation between vitamin D levels and the severity of periodontitis among 5,405 participants and revealed that the lower risk of severe periodontitis was associated with sufficient levels of vitamin D [68]. Hence, we recommend the evaluation of the serum vitamin D levels at the beginning of the periodontal therapy to enhance the treatment outcomes in vitamin D deficient patients. Restoring the normal levels of vitamin D in patients with deficiency through diet or supplementation may act as an effective way to decrease the disease progression and obtain a less tissue-destructive immune response. However, additional studies are needed to evaluate the extent to which vitamin D supplementation can enhance the outcomes of periodontal treatment [16, 17, 58].

We categorized the stages of periodontitis (stage I to IV) according to the 2017 classification of periodontal diseases. However, we recommend future longitudinal studies categorizing the extension of periodontitis according to the number of teeth involved in localized and generalized periodontitis. It will enhance measuring the progression of localized versus generalized periodontitis as well as the correlation with the clinical and biomedical markers as serum level of vitamin D. In addition, categorizing periodontitis into localized and generalized will show the different responses to periodontal treatment between both classes which will facilitate the development of more personalized treatment approaches for periodontitis.

Periodontal disease is linked to several common human diseases, including osteoporosis [69, 70]. Osteoporosis is a bone metabolism disease that results from a relative increase in bone resorption compared to bone formation, leading to a decrease in bone mineral density and destruction of bone microstructure, ultimately causing a series of osteoporotic fractures. Osteoporosis complicates periodontal disease, could be used as a risk indicator of periodontal disease development, and is associated with the progression of such disease [71–73].

Vitamin D is a regulator of overall calcium homeostasis, as well as a modulator of differentiation and activity of osteoblasts and osteoclasts. The active vitamin D compound stimulates the intestinal absorption of calcium and phosphate, mobilizes calcium and phosphate from bone, and promotes bone mineralization. Vitamin D is demonstrated to mediate bone metabolism directly through vitamin D receptors or indirectly through calcium and phosphate homeostasis [74, 75]. Deficiency in vitamin D leads to reduced bone mineral density and osteoporosis ultimately increasing the progression of periodontal diseases and causing resorption to occur in the jawbone [13]. The correlations among vitamin D, osteoporosis, alveolar bone destruction, and periodontal disease should not be overlooked by healthcare providers in dental clinics. These understandings can help periodontists predict the risk of periodontal disease in patients with alveolar bone destruction and osteoporosis, which will facilitate early diagnosis, prevention, or treatment of periodontal disease.

Production of the inflammatory cytokines in the periodontal tissues may be stimulated by microbial factors. IL-8 plays a significant role in the regulation of the inflammatory response through the activation of neutrophils and attraction of these cells to the area of periodontal inflammation [23, 24, 30]. Hence, evaluating the levels of these cytokines may help in the diagnosis of severity of the periodontal diseases. Therefore, we aimed to evaluate the levels of IL-8 in the study and control groups and to correlate their level with the severity of periodontitis. We found no significant changes in the levels of IL-8 between the study and the control groups. Moreover, we noticed that serum levels of IL-8 did not correlate with the severity of periodontitis among the study group.

A previous meta-analysis investigated the role of IL-8 in periodontitis and revealed no significant correlation between the risk of periodontitis and IL-8 polymorphism [30]. However, a different comprehensive analysis revealed that individuals with chronic periodontitis had greater levels of IL-8 protein and IL-8 gene expression than the healthy control group [40]. The contradictory results concerning the level of IL-8 in patients with periodontitis and healthy control may be due to several factors including the severity of periodontal diseases among the participants and the difference in the diagnostic and eligibility criteria. In addition, periodontitis is a multifactorial disease in which genetic and environmental factors can affect the severity and progression of the disease [76].

In conclusion, we comprehensively assessed the serum levels of vitamin D and IL-8 in patients with periodontitis and healthy controls. In addition, we aimed to assess the correlation of vitamin D and IL-8 levels to the severity and degrees of periodontitis by using the latest classification of periodontal diseases. The current study’s finding revealed a potential association between serum level of vitamin D with severity of periodontitis which necessitates screening vitamin D in patients with periodontitis. Future studies are endorsed to evaluate the causal effect of vitamin D deficiency and the onset and progression of periodontitis. In addition, similar studies are vital to assess periodontal health after vitamin D supplementation in periodontitis patients with vitamin D deficiency.

A key limitation of the current study is the case-control design which limits the ability to determine the causal relationship between serum levels of vitamin D and IL-8 and the occurrence or progression of periodontitis. In addition, the small sample size of this study limits the generalizability of our data to broader populations. So, we recommend future large sample-sized longitudinal studies in a large multiethnic population, with stepwise prospective follow-up and long-term clinical outcomes to confirm the directionality of these associations.

## Data Availability

The data that support the findings of this study are available from the corresponding author upon reasonable request.
